# Chronic Fatigue Syndrome – A clinically empirical approach to its definition and study

**DOI:** 10.1186/1741-7015-3-19

**Published:** 2005-12-15

**Authors:** William C Reeves, Dieter Wagner, Rosane Nisenbaum, James F Jones, Brian Gurbaxani, Laura Solomon, Dimitris A Papanicolaou, Elizabeth R Unger, Suzanne D Vernon, Christine Heim

**Affiliations:** 1 Division of Viral and Rickettsial Diseases, Centers for Disease Control and Prevention, Atlanta, GA, USA; 2 University of Toronto, Toronto, Canada; 3 Current Division of Parasitic Diseases, Centers for Disease Control and Prevention, Atlanta, GA, USA; 4 Department of Medicine, Emory University School of Medicine, Atlanta, GA, USA; 5 Merck & Co., Inc., Rahway, NJ, USA; 6 Department of Psychiatry and Behavioral Sciences, Emory University School of Medicine, Atlanta, GA, USA

## Abstract

**Background:**

The lack of standardized criteria for defining chronic fatigue syndrome (CFS) has constrained research. The objective of this study was to apply the 1994 CFS criteria by standardized reproducible criteria.

**Methods:**

This population-based case control study enrolled 227 adults identified from the population of Wichita with: (1) CFS (n = 58); (2) non-fatigued controls matched to CFS on sex, race, age and body mass index (n = 55); (3) persons with medically unexplained fatigue not CFS, which we term ISF (n = 59); (4) CFS accompanied by melancholic depression (n = 27); and (5) ISF plus melancholic depression (n = 28). Participants were admitted to a hospital for two days and underwent medical history and physical examination, the Diagnostic Interview Schedule, and laboratory testing to identify medical and psychiatric conditions exclusionary for CFS. Illness classification at the time of the clinical study utilized two algorithms: (1) the same criteria as in the surveillance study; (2) a standardized clinically empirical algorithm based on quantitative assessment of the major domains of CFS (impairment, fatigue, and accompanying symptoms).

**Results:**

One hundred and sixty-four participants had no exclusionary conditions at the time of this study. Clinically empirical classification identified 43 subjects as CFS, 57 as ISF, and 64 as not ill. There was minimal association between the empirical classification and classification by the surveillance criteria. Subjects empirically classified as CFS had significantly worse impairment (evaluated by the SF-36), more severe fatigue (documented by the multidimensional fatigue inventory), more frequent and severe accompanying symptoms than those with ISF, who in turn had significantly worse scores than the not ill; this was not true for classification by the surveillance algorithm.

**Conclusion:**

The empirical definition includes all aspects of CFS specified in the 1994 case definition and identifies persons with CFS in a precise manner that can be readily reproduced by both investigators and clinicians.

## Background

Chronic fatigue syndrome (CFS) affects 400,000 to 900,000 adults in the United States [1,2]. At least a quarter of those suffering from CFS are unemployed or receiving disability because of the illness; the average affected family forgoes $20,000 annually in lost earnings and wages (approximately half of the average United States household income); and the annual value of lost productivity in the United States is approximately $9 billion [2-4]. Despite the public health burden imposed by CFS, effective diagnostic, treatment and prevention strategies are not available because the etiology, risk factors and pathophysiology of CFS remain unknown (reviewed in [5]).

To some extent, the inconsistent and often conflicting results from studies of CFS reflect referral bias. Most published studies concerning CFS recruited patients from tertiary referral clinics. Patients receiving care for CFS are neither similar across clinics [6,7] nor necessarily representative of the population of people who suffer from CFS. Studies of CFS in the general population of Chicago have found that only 66% of people with CFS have consulted a physician concerning their fatigue and only 19% of them received a diagnosis of CFS [1]. Studies in the general population of Wichita have similarly reported that only 16% of individuals with the illness have been diagnosed by or treated for CFS by a physician [2].

In addition, the lack of consistent findings concerning the etiology, pathophysiology and risk factors for CFS reflects lack of standardized reproducible diagnostic criteria for CFS. CFS is defined as persistent or relapsing fatigue of at least 6-months' duration, that is not alleviated by rest, and that causes substantial reduction in activities. The fatigue cannot be explained by medical or psychiatric conditions and must be accompanied by at least 4 of 8 case defining symptoms (unusual post exertional fatigue, impaired memory or concentration, unrefreshing sleep, headaches, muscle pain, joint pain, sore throat and tender cervical nodes) [9]. An *International CFS Study Group *recently published recommendations concerning application of the case definition [10]. The *Group *recommended the use of validated instruments to obtain standardized measures of the major symptom domains of the illness. They specifically recommended: (1) the Medical Outcomes Survey Short Form-36 (SF-36), to measure functional impairment [11]; (2) a comprehensive instrument, such as the Checklist Individual Strength (CIS) [12] or the Multidimensional Fatigue Inventory (MFI) [13], to obtain reproducible quantifiable measures of fatigue,; and (3) the CDC Symptom Inventory to document the occurrence, duration and severity of the symptom complex [14]. We are aware of no published studies that have utilized this comprehensive battery of standardized clinically empirical instruments to characterize persons with CFS, persons with other unexplained fatiguing illnesses and non-fatigued controls.

The objective of the present study was to implement recommendations of the *International CFS Study Group *[10] and define CFS based on scores from standardized and validated instruments that assess the major dimensions of the illness specified by the 1994 CFS case definition [9]. Data came from a 2-day in-patient study of 227 people with CFS, with other chronically fatiguing illnesses, and matched non-fatigued controls identified in the general population of Wichita, Kansas. We used standardized and validated instruments to characterize the major dimensions of CFS – functional impairment (SF-36) – fatigue characteristics (MFI) – frequency and severity of accompanying symptoms (Symptom Inventory).

## Methods

### Study design

This study adhered to human experimentation guidelines of the U.S. Department of Health and Human Services and the Helsinki Declaration. The CDC Institutional Review Board approved the study protocols (IRB #3504). All participants were volunteers who gave informed consent.

### Recruitment

The study was conducted from December 2002 to July 2003 and enrolled participants, younger than 69 years from the 1997 through 2000 *Wichita CFS Surveillance Study *[2,3,15]. In brief, the *Surveillance Study *followed a cohort of 7,162 fatigued and non-fatigued adults representative of the Wichita population at 12-, 24-, and 36-month intervals with telephone interviews and clinical evaluations.

Two hundred twenty-seven people enrolled in the clinical study. We invited the 70 people classified as CFS at least once during the 4-year *Surveillance Study*; 58 (83%) agreed to participate. Each CFS case was matched to a control based on sex, race/ethnicity, age and body mass index. Matched-controls were selected from the cohort who participated in surveillance at the baseline and all follow-up periods, who did not have medical or psychiatric exclusions, and who had not reported fatigue of at least 1-month duration; 55 controls participated. We also invited 70 (randomly selected) of the 158 *Surveillance Study *participants identified with unexplained chronic fatigue that did not meet the criteria for CFS; 59 (84%) agreed to participate. We term this group 'insufficient symptoms or fatigue' (ISF) and discuss it below. Finally, although melancholic depression is considered exclusionary for CFS, we invited all 41 surveillance participants who met the criteria for CFS except for concurrent melancholic depression and all 39 with ISF and melancholic depression; 27 (66%) and 28 (72%), respectively, enrolled in the study.

### Clinical assessment

#### Medical evaluation

Those who agreed to participate were admitted to a Wichita hospital research unit for two days. Hospital staff was unaware of subjects' enrollment status, as were the subjects. To identify exclusionary medical conditions [9,10], subjects provided a standardized past medical history and review of systems, which they completed at home. During admission, a study nurse reviewed this and resolved missing items and patients' questions. Study participants brought all current prescribed and over the counter medications and dietary supplements to the hospital. These were reviewed and catalogued by a study nurse. At the time of admission, subjects underwent a standardized physical examination conducted by a specifically trained physician. The physician also reviewed past medical history, review of systems and medications; then, following the standardized physical examination, she evaluated specific systems in more detail, as warranted. Finally, patients provided blood and urine for routine analyses; the study physician reviewed results the following morning and considered them in terms of the previous evening's physical examination. Medical conditions exclusionary for CFS followed 1994 case definition criteria as modified by the *International CFS Study Group *[9,10].

#### Psychiatric evaluation

Licensed and specifically-trained psychiatric interviewers administered the Diagnostic Interview Schedule (DIS) to identify current DSM-IV Axis I psychiatric disorders [16]. Exclusionary psychiatric illnesses included: current melancholic depression; current or lifetime bipolar disorder or psychosis; substance abuse within two years and eating disorders within 5 years [9,10]. Psychiatric disorders considered non-exclusionary included depression, anxiety disorders and somatoform disorders.

#### Evaluation of functional impairment – fatigue – accompanying symptoms

Subjects also completed the SF-36 [11], the MFI [13] and the Symptom Inventory [14] before arriving at the hospital, at which time the study manager, who resolved missing responses, reviewed them. The SF-36 assesses functional impairment in 8 areas: limitations in physical activities (physical function), limitations in usual role activities because of physical health problems (role physical), limitations in usual role activities because of emotional problems (role emotional), bodily pain, general health perceptions (general health), vitality (energy and fatigue), social function and general mental health. Scores in each area reflect function and wellbeing and lower values indicate more impairment. The MFI assesses general fatigue, physical fatigue, mental fatigue, reduced motivation and reduced activity. The score in each dimension reflects fatigue severity and higher values indicate more severe fatigue. Finally, the Symptom Inventory assesses the occurrence, frequency and intensity of 19 symptoms over the preceding month. For this study, we used the Symptom Inventory Case Definition Score defined as the sum of the product of the frequency and intensity scores of the 8 CFS case defining symptoms [14].

### Illness classification

Because study participants had been classified as CFS, ISF or melancholic depression during surveillance up to 6 years previously and because CFS is cyclic in the occurrence and severity of its symptoms, we classified participants' illness status at the time they participated in the clinical study. We used two different schemes based on 1994 CFS case definition criteria to classify them.

#### Illness classification by surveillance criteria

We applied the 1994 case definition algorithm as it had been used during surveillance to classify subjects. They completed a questionnaire concerning their illness, it was reviewed upon arrival at the hospital, and omissions were rectified. Fatigue fulfilling the case definition was defined as duration longer than 6 months; **and **not relieved by rest (based on a response "a little or not at all" to "Is your fatigue relieved by Rest?"); **and **caused substantial reduction in occupational, educational, social, or recreational activities (based on a response "a lot" to "Does fatigue interfere with...?"). The presence of ≥ 4 of the 8 case-defining symptoms was determined by tabulating positive responses ("all of the time or most of the time") to queries concerning the 8 case-defining symptoms (e.g. "During the past month how often have you had a sore throat?"). Fatigued subjects not meeting fatigue or not meeting symptom criteria were classified as insufficient symptoms or fatigue (ISF). Subjects recruited because they were CFS or ISF during surveillance but who did not report fatigue at the time of the clinical study were considered in remission.

#### Illness classification by standardized clinically empirical criteria

We used information from the SF-36, MFI and Symptom Inventory to classify subjects empirically according to the 3 main dimensions of CFS: functional impairment (SF-36), fatigue (MFI) and accompanying symptoms (Symptom Inventory). We defined substantial reduction in occupational, educational, social, or recreational activities as scores lower than the 25th percentile of published US population [11] on the physical function (≤ 70), **or **role physical (≤ 50), **or **social function (≤ 75), **or **role emotional (≤ 66.7) subscales of the SF-36. We defined severe fatigue as ≥ medians of the MFI general fatigue (≥ 13) **or **reduced activity (≥ 10) scales. Finally, subjects reporting ≥ 4 symptoms **and **scoring ≥ 25 on the Symptom Inventory Case Definition Subscale were considered to have substantial accompanying symptoms.

Subjects who met all 3 criteria (SF-36 **and **MFI **and **Symptom Inventory) when they entered the clinical study were classified as CFS according to standardized clinically empirical criteria; those who met some but not all 3 criteria were considered as insufficient symptoms or fatigue (ISF); those who met none of the criteria were classified as Not Ill.

### Statistical analyses

Because classification as CFS at the time of the clinical study was not completely concordant with recruitment classification, matching was not maintained. However, cases and controls were demographically comparable.

We used the χ^2^-test to compare proportions. We performed 2-step cluster analysis using log-likelihood distance measures to determine if subjects fell into different groups according to the SF-36, MFI and Symptom Inventory scales. Differences among fatigue subgroups with respect to the SF-36, MFI and Symptom Inventory scales were assessed by one-way analyses of variance followed by Tukey's pair-wise multiple comparisons method (equal group variances) or Dunnett's T3 procedure (unequal variances) at a significance level of 0.05. To measure correlations between the SF-36 subscales, MFI subscales and Symptom Inventory, we used the Pearson correlation coefficient. We utilized SPSS 11.5 (SPSS Inc., Chicago, IL) for all analyses.

We used an algorithm developed in Mathematica 5.0 (Wolfram Research, Champaign, IL) to assess the likelihood of global characteristics of the data (the code is available upon request). The algorithm was based on principal components analysis and a Monte Carlo method for computing the mean and variance of the likelihood. Information entered into the algorithm included a matrix of scores for a nominal group (Not Ill) against which other groups (e.g. CFS, ISF) were compared, and the minimum difference in SF-36 and MFI scores between the nominal and other groups. The matrix was divided into its principal components so that correlations between the various measurements were accounted for. Simulations based on the statistical characteristics of the matrix were performed and the likelihood of seeing a consistent ascending or descending pattern between two or more groups for all of the SF-36 and MFI scores was evaluated.

## Results

### Enrolment characteristics

Two hundred twenty-seven people participated in the study and participation rates were similar among the fatigue categories (p = 0.26). Participants' median age was 51 years (range 25 – 69), 81.9% were women, 94.3% were white, and 79.3% were overweight or obese (median body mass index = 29, range 16 – 40). Demographic characteristics did not vary significantly according to recruitment classification (Table 1).

**Table 1:** General characteristics of subjects by recruitment classification.

	CFS (n = 58)	CFS-MDDm (n = 27)	ISF (n = 59)	ISF-MDDm (n = 28)	Control (n = 55)	All (N = 227)
% Women	86.2	77.8	71.2	92.9	85.5	81.9
Median age (years)	51.5	48.0	50.0	51.5	51.0	51
% White	94.8	92.6	93.2	92.9	96.4	94.3

BMI (%)						
<18.5: Underweight	1.7	0.0	1.7	0.0	1.8	1.3
18.5–24.9: Normal	13.8	33.3	20.3	21.4	16.4	19.4
25–29.9: Overweight	41.4	29.6	32.2	35.7	36.4	34.7
30–34.9: Obese	31.0	29.6	35.6	21.4	34.5	31.7
≥35: Morbidly Obese	12.1	7.4	10.2	21.4	10.9	11.9

### Medical and psychiatric exclusions

We identified 29 people with newly documented medical conditions considered exclusionary because they could account for fatigue and accompanying symptoms [9,10]. Medical exclusions included active and inadequately treated thyroid disease (n = 6), neurological disease (n = 6), inflammatory disease (n = 4), C-reactive protein levels 2 to 4 times normal (n = 3), severe anemia (n = 2), uncontrolled diabetes (n = 2), cardiac disease (n = 2), renal disease (n = 2), breast cancer post-treatment (n = 1) and liver disease (n = 1). We also identified 4 persons with exclusionary psychiatric conditions; 3 had bipolar disorders (1 person with a bipolar disorder was also medically excluded because of severe anemia), and 1 alcohol abuse. Finally, 5 subjects could not be classified because DIS data were missing. These 37 people were not considered further in this report.

Twelve fatigued and two control subjects (among the remaining 190 participants) presented with newly identified melancholic depression. However, 34 (73.9%) of the 46 subjects enrolled because they had been classified during the surveillance study as melancholic depression no longer had evidence of the condition. Following recommendations of the *International CFS Study Group*, only current MDDm was considered exclusionary for CFS. Thus, the remainder of this paper discusses only the 164 participants with no medical or psychiatric exclusionary conditions.

### Illness classification by surveillance criteria

When we applied the same case definition algorithm used in the surveillance study to classify subjects at the time they entered this clinical study only 16 had a current classification of CFS, 76 of ISF, 48 were not fatigued controls, and remission was identified in 24 recruited as CFS/ISF who no longer reported fatigue (Table 2). Most (87%) of the 46 subjects enrolled because they were considered CFS during surveillance did not meet the same case definition criteria at the time of the clinical study: most (58.7%) were classified as ISF and 10.9% were in remission. This difference was less striking among those recruited as ISF: 64.6% retained their ISF classification; but 18.8% were in remission and 8.3% were assigned a current classification of CFS.

**Table 2:** Recruitment and Current Classifications of 190 Subjects; 37 participants with medical or psychiatric exclusions other than melancholic depression excluded.

**Current Classification by Surveillance Criteria**						
**Classification During Surveillance**	CFS	ISF	Remission	Control	Exclusions	

CFS	6 (13.0)	27 (58.7)	5 (10.9)	0	8 (17.4)	**46**
ISF	4 (8.3)	31 (64.6)	9 (18.8)	0	4 (8.3)	**48**
Control	0	0	0	48 (96.0)	2 (4.0)	**50**
CFS MDDm	5 (23.8)	9 (42.9)	2 (9.5)	0	5 (23.8)	**21**
ISF MDDm	1 (4.0)	9 (36.0)	8 (32.0)	0	7 (28.0)	**25**
	**16**	**76**	**24**	**48**	**26**	**190**

Columns show number of subjects and (percent of row)

This lack of agreement between diagnosis during surveillance and diagnosis by surveillance criteria at the time of the in-hospital study could reflect the cyclic nature of CFS and changes over time. So we used an independent approach to evaluate the current overall severity of illness. To do this we applied a 2-step cluster analysis to scores from the 8 SF-36, 5 MFI and Symptom Inventory Case Definition scale scores. These 14 interrelated scores reflect overall severity of illness and 3 distinct clusters emerged (Table 3). The most severely ill subjects, with the lowest SF-36, highest MFI and highest Symptom Inventory scores were in cluster-1. Cluster-3 included the least severely ill subjects, whose scores essentially reflected population norms. Cluster-2 represented intermediate illness severity. Most (89.6%) of the non-fatigued controls were in the least severely ill cluster, but 5 controls (10.4%) were in the intermediate severity cluster; CFS, ISF and remission were spread across all 3 clusters (Table 4).

**Table 3:** Characteristics of 2-steps 3-cluster analysis solution for 164 subjects with no medical or psychiatric exclusions.

**Characteristic**	**Cluster 1 **Most Severe (n = 30)	**Cluster 2 **Intermediate (n = 67)	**Cluster 3 **Least Severe (n = 67)
**SF-36 Scales (mean ± sd)**			
Physical function	49.5 ± 21.8	71.8 ± 20.1	91.0 ± 10.3
Role physical	12.5 ± 26.9	48.1 ± 36.0	92.5 ± 19.5
Bodily pain	39.5 ± 18.3	53.7 ± 16.5	79.4 ± 15.6
General health	45.4 ± 19.3	67.4 ± 13.8	85.3 ± 12.6
Vitality	14.3 ± 11.0	34.6 ± 15.8	69.6 ± 16.0
Social function	45.4 ± 24.0	67.9 ± 18.4	96.3 ± 8.7
Role emotional	47.8 ± 41.7	74.1 ± 35.2	96.0 ± 13.6
Mental health	61.5 ± 19.5	74.4 ± 13.5	86.9 ± 9.1
**MFI Scales (mean ± sd)**			
General fatigue	18.4 ± 1.4	15.1 ± 2.7	8.7 ± 3.2
Physical fatigue	15.4 ± 2.7	11.6 ± 2.8	6.7 ± 2.1
Mental fatigue	15.9 ± 3.5	10.3 ± 3.9	6.6 ± 2.7
Reduced motivation	13.4 ± 4.0	10.3 ± 3.0	6.2 ± 2.0
Reduced activity	15.8 ± 3.3	11.9 ± 3.5	5.9 ± 2.0
**Symptom Inventory (mean ± sd)**	52.2 ± 24.8	21.6 ± 12.6	7.0 ± 8.5

All clusters were significantly different from the other clusters with respect to all scales

**Table 4:** Current classification and illness severity by cluster analysis of 164 study participants with no medical or psychiatric exclusions.

**Cluster Group**				
**Current Classification Surveillance Criteria**	1 Most Severe	2 Intermediate	3 Least Severe	

CFS	7 (43.8)*	9 (56.3)	0	**16**
ISF	16 (21.1)	55 (72.4)	5 (6.6)	**76**
Remission	1 (4.2)	13 (54.2)	10 (41.7)	**24**
Control	0	5 (10.4)	43 (89.6)	**48**
	**24**	**82**	**58**	**164**

* percent of row

### Illness classification by standardized clinically empirical criteria

Finally, we classified study participants by standardized clinically empirical criteria and found little agreement within subjects according to their classification at the time of this study by surveillance criteria (Table 5). To explore the characteristics of people classified by the standardized clinically empirical criteria, we evaluated associated SF-36, MFI and Symptom Inventory scores.

**Table 5:** Illness classification at the time of the clinical study by surveillance criteria and by standardized clinically empirical criteria of 164 study participants with no medical or psychiatric exclusions.

**Current Classification Surveillance Criteria**	CFS	ISF	Not Ill	
CFS	10 (62.5)	6 (37.5)	0	**16**
ISF	32 (42.1)	38 (50.0)	6 (7.9)	**76**
Remission	1 (4.2)	13 (54.1)	10 (41.7)	**24**
Control	0	4 (8.3)	44 (91.7)	**48**
	**43**	**61**	**60**	**164**

Columns show number of subjects and (percent of row)

#### SF-36

Persons with fatiguing illnesses showed significantly more functional impairment (lower scores) in all 8 scales of the SF-36 compared to the Not Ill (Table 6). Those with CFS had significantly lower scores on all SF-36 subscales than those with ISF (all post-hoc-comparisons: p < 0.001; except role-emotional p = 0.027 and mental health p = 0.050).

**Table 6:** Medical Outcomes Study Short-Form 36 (SF-36), Multi-Dimensional Fatigue Inventory (MFI) and Symptom Inventory Scores according to current illness classified by standardized clinically empirical criteria in 164 participants with no exclusionary medical or psychiatric conditions.

**Scale**	CFS	ISF	Not Ill
**SF-36**			
Physical function^+^	53.3 ± 21.5 *	**77.5 ± 18.9**	89.7 ± 13.2
Role physical^+^	18.0 ± 28.5 *	**60.7 ± 37.2**	88.8 ± 25.0
Bodily pain	41.7 ± 15.7 *	**59.7 ± 20.0**	77.9 ± 16.0
General health	51.2 ± 20.4 *	**70.1 ± 13.6**	85.2 ± 13.3
Vitality	18.6 ± 12.5 *	**37.3 ± 18.5**	72.3 ± 13.0
Social function^+^	50.0 ± 22.7 *	**74.2 ± 19.6**	94.8 ± 10.6
Role emotional^+^	55.8 ± 42.2 *	**76.5 ± 33.5**	96.1 ± 13.8
Mental health	66.4 ± 18.5 *	**74.6 ± 14.0**	87.5 ± 9.0
**MFI**			
General fatigue^+^	18.5 ± 2.1 *	**14.9 ± 2.7**	8.0 ± 2.6
Physical fatigue	14.1 ± 3.2 *	**11.0 ± 3.2**	6.8 ± 2.2
Mental fatigue	14.1 ± 4.2 *	**10.0 ± 4.3**	6.6 ± 2.6
Reduced motivation	12.2 ± 3.8 *	**10.1 ± 3.3**	6.1 ± 2.0
Reduced activity^+^	14.7 ± 3.1 *	**11.5 ± 4.0**	5.6 ± 1.6
**Symptom Inventory**^+^	47.3 ± 22.0 *	**18.6 ± 11.9**	6.1 ± 7.1

^+^Indicate subscales used in validated clinically empirical CFS case definition

Scores are mean ± standard deviation

Bolded figures indicate that specific group scores are significantly different (p < 0.05) from non-fatigued scores.

* indicates that CFS scores are significantly different from ISF scores

Note: Low scores in the SF-36 indicate more severe conditions, whereas in the MFI higher scores indicate more severe conditions.

#### MFI

Persons with fatiguing illnesses had significantly higher scores on all scales (indicating more severe fatigue) than Not Ill (Table 6). Consistent with the SF-36, those with empirically defined CFS had significantly higher scores on all MFI subscales than subjects with ISF (all post-hoc-comparisons p < 0.001; except reduced motivation p = 0.016).

#### Symptom inventory

The Symptom Inventory reflected similar associations (Table 6). Fatigued persons had significantly higher Symptom Inventory scores than the Not Ill (p < 0.001) and subjects with CFS had significantly higher scores than those with ISF (post-hoc-comparison p < 0.001).

#### Monte Carlo analysis

We used a Monte Carlo analysis to assess the likelihood of seeing this global pattern of significant differences between empirically defined CFS and ISF and Not Ill on all scales of the SF-36, MFI and Symptom Inventory. The probabilities that each group would separate from the others by such a wide margin on all scales by chance alone were vanishingly small (p < 10^-7^). Even the consistent difference between the Not Ill and ISF groups on all 14 scores was extremely unlikely (p < 0.0001). This likelihood was great enough, however, that up to 10 of the not fatigued people from the screening sample (56,000) could have been clustered with the ISF group due to random variation within the Not Ill population (given the mean plus 3 standard deviations in the estimate of the variance on all scales from the Not Ill population). That is, some of the movement (Table 5) from control to ISF, and likewise ISF to CFS, could have resulted from classification of individuals at the extreme end of the bell curve of one population as being members of a different population with a different illness state.

## Discussion

The primary objective of this study was to implement recommendations of the *International CFS Study Group *[10] and define CFS on the basis of scores from standardized and validated instruments that assess the major dimensions of the illness as specified by the 1994 CFS case definition [9]. Functional impairment, fatigue and an accompanying symptom complex characterize CFS. We defined functional impairment as scores ≤ 70 on the physical function **or **≤ 50 on the role physical **or **≤ 75 on the social function **or **≤ 66 on the role emotional subscales of the SF-36. We defined severe fatigue as scores ≥ 13 on the general fatigue **or **≥ 10 on the reduced activity subscales of the MFI. Finally, we defined the accompanying symptom complex as reporting the occurrence of ≥ 4 of 8 symptoms **and **scoring ≥ 25 on the Symptom Inventory Case Definition subscale. One could debate our choice of specific subscales from the SF-36 and MFI and the specific cut-off values we chose on the SF-36, MFI and Symptom Inventory. However, these instruments have been validated, have been used extensively in studies of CFS and other illnesses, and are known to be reproducible. In contrast, most studies of CFS merely note that they used the 1994 case definition and they do not generally specify how disability, fatigue and symptom occurrence were elucidated. Thus, it is difficult to assess the validity of their diagnostic criteria and essentially impossible to compare results between studies critically.

This study showed scant stability of CFS over time, when diagnosed by the usual algorithm (based on patients' subjective responses to direct questions as to whether they feel fatigued, if they perceive their fatigue causes substantial reduction in daily activities, and whether at least 4 case defining symptoms are present). There was poor correlation between illness classification during surveillance (recruitment classification) and classification by the same criteria during the clinical study. While this might reflect fluctuation in illness over time, illness categories (CFS, ISF, Remission, non-fatigued) defined by this surveillance classification scheme were not consistent with respect to overall illness severity. In contrast, when we defined illness categories (i.e. CFS, ISF, Not Ill) according to validated clinically empirical instruments, we found an extremely strong relationship between diagnostic category and every measure of severity. It is not surprising that scores on the 4 SF-36 and 2 MFI scales used in classification were significantly correlated with diagnosis, but the 4 SF-36 and 3 MFI scales not used in classification showed similar significant associations with diagnostic category. Although correlated, the dimensions of impairment and fatigue defined by these instruments represent statistically independent factors. Finally, while only 13% of patients who met the 1994 case definition criteria during surveillance met those same criteria in the present study, 40% fulfilled CFS criteria of the clinically empirical definition. Thus, the clinically empirical case definition may be less affected by the day-to-day fluctuation of the illness and rather reflect the underlying chronic illness process.

Defining CFS in this empirical manner will improve the precision of case ascertainment in research studies, it will provide a standard reproducible means of following the clinical course over time, and it will help to clarify the extent to which patients from different referral clinics are similar (or dissimilar). Finally, this strategy can be used in primary care settings and will give health care providers a standard and reproducible method for diagnosing CFS. The SF-36, MFI and Symptom Inventory are relatively short forms that are completed by patients: scoring is straightforward and can be accomplished by clinic staff. The MFI and Symptom Inventory are in the public domain but the SF-36 is sold under license.

Use of this diagnostic strategy also provides health care providers with objective measures of the disability associated with CFS. The SF-36 measures functional impairment in 8 distinct dimensions. Normative values have been rigorously derived and documented in each dimension for healthy individuals and in a wide variety of disease states [11,17,18]. For example, in this study subjects with empirically defined CFS had lower scores on all scales, except physical function and general health, than those of patients with congestive heart failure. In addition to documenting impairment, scores on individual components of the SF-36 and MFI can be used to identify specific aspects of the illness for specific interventions and changes in scores over time can be used to monitor response to therapeutic interventions.

In addition, there is a certain mathematical appeal to the new criteria. Principal components analysis of the matrix of 14 scores (8 SF-36, 5 MFI, and the Symptom Inventory Case Definition subscale) for those enrolled in this study showed that their symptoms define a roughly 3-dimensional space (rather than 14) and the first 3 principal components accounted for 76% of the total variation among individuals. These 3 components roughly align with the most significant scales of the SF-36, MFI and Symptom Inventory, all of which are part of the 3 inequalities we used to empirically classify illness. As a consequence, the 3 components cleanly separate the empirically classified groups of CFS, ISF and Not Ill in 3-dimensional space, whereas this separation could not be achieved with the older classification scheme

Finally, our findings highlight the importance of continued evaluation of persons with CFS. Clinical management of CFS poses a difficult challenge for health care providers, patients and patients' families/associates because of the absence of physical signs and because illness severity and symptoms vary dramatically over time. Not all new symptoms or worsening of severity should be attributed to CFS a priori. Thirty-two (13%) of the study participants had serious medical or psychiatric diseases diagnosed for the first time during this study and another 18 (7%) were newly discovered to have melancholic depression, thus supporting inclusion of this disease in the exclusionary criteria. Those 49 subjects had been repeatedly evaluated during the Wichita surveillance study and had no prior evidence of such conditions.

## Conclusion

In conclusion, we found that CFS can be defined by use of readily available instruments that evaluate the major components specified in the 1994 case definition. People with CFS defined in this manner are clinically distinct from those with unexplained fatigue (i.e. ISF) that does not meet the criteria for CFS. Future research studies should define CFS in this manner and this diagnostic strategy can also be used for the clinical evaluation and subsequent follow-up of patients with CFS.

## Competing interests

The author(s) declare that they have no competing interests.

## Authors' contributions

WCR conceived of the study, served as principal investigator throughout its execution and wrote the manuscript; DW conceived of the scale to summarize symptom impact, collaborated in data analysis and writing the manuscript; RN collaborated in study design, had primary responsibility for statistical analysis, and collaborated in writing the manuscript; JFJ collaborated in the clinical study and in preparation of the manuscript; BG collaborated in data analysis and interpretation, conceived the concept of PCA-MC analysis to assess the likelihood of global patterns appearing in the data, and collaborated in writing the manuscript; LS participated in fieldwork, collaborated in analysis and interpretation of the data and writing the manuscript; DAP collaborated in study design, participated in fieldwork, collaborated in analysis and interpretation of the data and writing the manuscript; ERU was instrumental in the conception and design of the study, collaborated in interpretation of the data and writing the manuscript; SDV was instrumental in the conception and design of the study, collaborated in interpretation of the data and writing the manuscript; CH was instrumental in the conception and design of the study, participated in fieldwork, collaborated in analysis and interpretation of the data and writing the manuscript.

## Pre-publication history

The pre-publication history for this paper can be accessed here:


